# Virtual patients: the influence of case design and teamwork on students’ perception and knowledge – a pilot study

**DOI:** 10.1186/1472-6920-14-137

**Published:** 2014-07-08

**Authors:** Frederik Jäger, Martin Riemer, Martin Abendroth, Susanne Sehner, Sigrid Harendza

**Affiliations:** 1 Department of Computational Neuroscience, University Hospital Hamburg-Eppendorf, Germany, Martinistr. 52, 20246 Hamburg, Germany; 2 Department of Internal Medicine, University Hospital Hamburg-Eppendorf, Martinistr. 52, 20246 Hamburg, Germany; 3 Institute for Biometrics and Epidemiology, University Hospital Hamburg-Eppendorf, Martinistr. 52, 20246 Hamburg, Germany

**Keywords:** CASUS, E-learning, Teamwork, Virtual patients

## Abstract

**Background:**

Virtual patient (VP) cases are an effective teaching method, although little is known about how to design and implement them for maximum effectiveness. The aim of this study was to explore the effect of case design and teamwork on students’ learning outcome.

**Methods:**

One hundred forty-six undergraduate medical students participated in a mandatory medical computer science course consisting of five seminars. At the end of each seminar, they worked on one VP case, either in teams of two or individually. Each student filled out an introductory and a final survey and a feedback sheet after completing each case. Additionally, there was a surprise multiple choice (MC) test after the last seminar with three questions regarding each case.

**Results:**

Students with more clinical experience and students who had worked in a team performed significantly better on MC questions. Students with less clinical experience more frequently used information which had been positioned less prominently on the case material. Certain aspects of case design were rated more positively by students who had an interest in e-learning. In general, students preferred to work on cases for less than 15 minutes.

**Conclusions:**

Clinically more advanced students and students working with a partner seem to benefit most from short VP cases with prominently presented information.

## Background

In times of digitalization and ubiquitous internet connections, the use of computer-based media is establishing itself as an ever-growing domain in medical education [1]. E-learning allows access that is not limited by time constraints. Furthermore, interactive learning has become an integral part of education at many medical schools [2]. It has also been demonstrated that e-learning can be just as effective as conventional teaching methods [3]. A growing branch of e-learning is the use of virtual patients (VPs). VPs are becoming particularly popular for teaching clinical reasoning [4], due to the interactive learning experience, which can simulate some of the diagnostic steps and the clinical decision-making processes of physicians’ daily work. Additionally, they have been shown to be effective at propagating knowledge [5].

Many different approaches and programs for designing VP cases, including different structures and layouts, are currently available [6]. The CASUS platform, for instance, is a system for ‘multimedia paper cases’, which provides a basis for using cases in different countries and languages [7]. This is advantageous as it has been suggested that a simple and easily accessible design of the e-learning platform can improve results when working with VPs [8].

In order to optimize the effectiveness of VP cases, design and implementation play an important role. Regarding implementation, it seems that relevance to seminars and corresponding tests [9], as well as a smooth, balanced [10] and functional integration into the curriculum [11], are important in order to motivate students to actually use the cases. For case design, a focus group study conducted by Huwendiek et al. [12] identified important aspects to consider and offered suggestions on how to optimize the design of VPs, also stating that these findings could be followed up in quantitative studies.

Another important aspect is teamwork, which has been shown to activate learners and to enhance knowledge [13] and, therefore, might also be relevant for learners’ motivation and retention of knowledge when working with VPs. In a study by Edelbring et al. [14], students reported a perceived benefit to their clinical reasoning skills from working on VP cases with a partner and discussing their patient management approaches. Furthermore, motivation has been identified as being a dependent variable influenced by autonomy [15], which is also a necessary skill when working on VP cases. However, in one survey among students working with VPs, up to 86% of the participants preferred to work in teams or at least had no preference towards individual work [16].

With this study, we attempted to identify aspects of case design, as well as aspects of working with VP cases, which might affect students’ perception of and learning success with VP cases. We designed and implemented five VP cases, where three main design aspects (narrative style, question type, question content) from the literature [12] were modified between cases, and students either worked alone or with a partner in order to answer the following research questions: Is a particular version of these three design aspects of VP cases favoured by students? Does working on a VP case together with a partner result in a better learning outcome? We hypothesize that certain aspects of VP case design are favoured by students and that teamwork will lead to greater retention of knowledge.

## Methods

### Virtual patients

Five patient cases were created using the e-learning authoring system CASUS [17], which offers a flashcard-style interface. Each card supports a text body, a media element (e.g. for radiographs, lab results or short videos), an interactive question, and an answer comment, as well as an additional ‘expert comment’. This design offers the opportunity to have optional, in-depth information based on the user’s needs: Terms and abbreviations can be explained through ‘mouseover’ pop-ups, hyperlinks can provide further information, answer comments explain what is correct or important and the ‘expert comment’ offers an overview or specific details on certain topics. Each case consisted of six to nine interactive cards and was designed for students to complete within 30 minutes time, when working rigorously. All cases were based on the files of real patients and featured the following diseases: hepatitis (case 1), pneumothorax (case 2), hypocalcaemia (case 3), mechanical ileus (case 4) and vitamin B12 deficiency (case 5). Cases 1 and 2 are given as samples in Table 1. For our case design, we used a list of ten criteria postulated to be important for creating a good e-learning case in a qualitative study [12]. While we evenly adhered to most design criteria (as far as the CASUS platform allowed) throughout the cases, we picked three aspects to be presented in two variations: ‘focus on relevant learning points’, ‘authenticity of student task’, and ‘questions and explanations to enhance clinical reasoning’. For each of these three aspects, one variation was in line with the criteria, while the other deviated. To test ‘focus on relevant learning points’, we varied the amount of irrelevant text (i.e. ‘narrative style’). For ‘authenticity of student task’ we either had the students write their answers as free text or answer MC questions (i.e. ‘question style’). For ‘questions and explanations to enhance clinical reasoning’ we either asked procedural questions or mere knowledge questions (i.e. ‘question type’). Each case contained one variant of each design aspect and the mix was assigned so that no combination of the three aspects occurred twice. For the case feedback sheets, we designed questions to measure how much the students favoured a certain variant. We included general questions regarding ‘relevance’, ‘appropriate use of media’, ‘specific feedback’ and ‘recapitulation of key learning points’ in the study in order to gauge the importance and approval of those aspects. The remaining aspects ‘appropriate level of difficulty’, ‘interactivity’ and ‘authenticity of the web-based interface’ were only subject to indirect observation.

**Table 1:** Case examples

**Aspects**	**Case 1: hepatitis**	**Case 2: pneumothorax**
Description	A patient returns from a trip to Africa with symptoms of an infection.	A passenger on an international flight experiences acute chest pain.
Suggested study time	30 min	30 min
Objectives and outcomes	Students will learn the differential diagnoses, relevant lab results and serum tests for hepatitis.	Students will learn the first measures for dealing with acute chest pain, as well as causes, symptoms, diagnostics and treatment of a spontaneous pneumothorax.
Perspective	Case is told from the point of view of a single treating physician.	Case is told from the point of view of a single treating physician.
Narrative style	Patient is presented using precise descriptions and condensed information.	The case is told like a story, describing the setting and background of the situation beyond necessary information.
Media	Pictures of physical findings (e.g. jaundice), tables of lab results	Radiology findings, videos on how to place a chest tube
Interactivity use	Nine MC questions, some with multiple correct answers. ‘Mouseover’ explanations, hyperlinks and expert comments	Eight textboxes to answer questions and compare with the suggested answers. ‘Mouseover’ explanations, hyperlinks and expert comments
Question content	Facts about the disease, lab results and differential diagnoses; can be answered without reading the case	Diagnostic steps, interpreting findings, how to treat the patient; details which the treating physician would think about in that situation
Path type	Linear (string of pearls)	Linear (string of pearls)
Feedback use	Explanations regarding the correctness/incorrectness of each MC option	Reference answers and explanations for the users to compare to their own input
Expert comment	Detailed information on jaundice and hepatitis B serology	Video instructions on placing a chest tube, X-ray studies of pneumothorax

### Questionnaires

**Figure 1:**
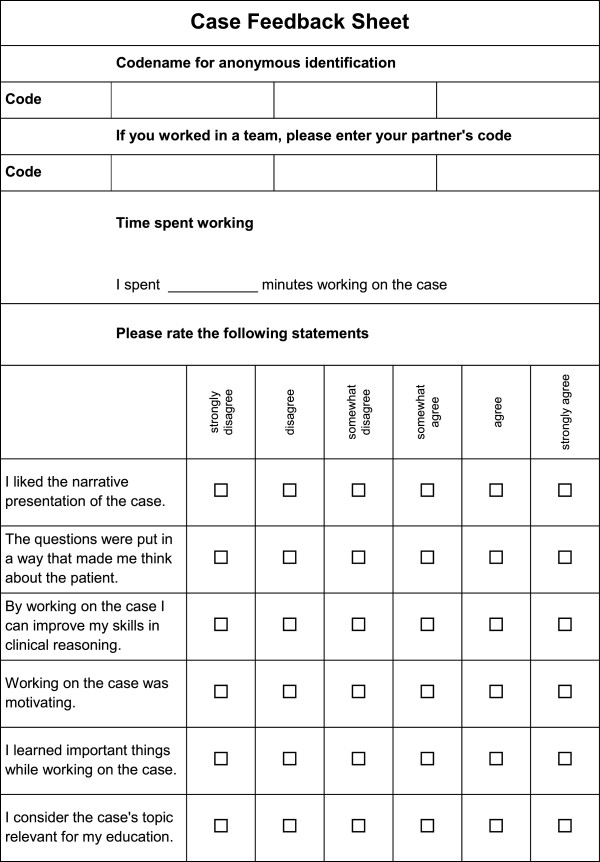
Case feedback sheet.

Three types of questionnaires were developed: an introductory survey, a feedback sheet for each case and a final survey with an added 15 question multiple choice test. All questionnaires used a 6-point Likert scale (1 being the lowest value and 6 being the highest value), as well as dichotomous questions and questions with multiple selections. The introductory survey included sociodemographic questions regarding gender, age, current semester, experience with and attitude towards e-learning. The questionnaires were developed by the authors during a brainstorming discussion with the aim of defining factors of possible influence on working with VPs. With the feedback sheet (Figure 1) for each case, students were asked whether they worked alone or with a partner, how long they worked on the case and whether they completed it. They were also asked to rate statements regarding the design of the case and their experience working on it on a Likert scale. This questionnaire was developed to assess the changes in case design elements based on the elements for VP cases described by Huwendiek et al. [12]. In the final survey, students were asked about their preferred amount of time to work on an e-learning case and several questions on qualitative aspects of the case format and how they worked with it. These questions were developed in order to measure further aspects regarding the cases. None of the questionnaires were validated. In addition, students had to unexpectedly answer 15 multiple choice questions, three on the topic of each case. From five answers the only correct or only incorrect answer had to be chosen. Questions only included content provided by the five cases. A sample MC question is given in Table 2.

**Table 2:** Example MC question

**Please interpret the following test results: Anti-Hbc-antibodies: positive; anti-Hbs-antibodies: positive; HBs-antigen: negative**	
(A)	No final conclusion can be made without knowing the patient’s Anti-Hbe status
(B)	Acute hepatitis B infection
(C)	Chronic hepatitis B infection
(D)	Status after hepatitis B vaccination
(E)	Status after hepatitis B infection*

*correct answer.

### Study design and participants

At the Hamburg Medical School, the third, fourth, and fifth years of a six-year medical curriculum include seven different modules of 12 weeks each, which can be completed by the students in any order they choose. Hence, students’ knowledge and experience levels can widely vary depending on the number of modules they have already finished. In October 2012, 146 students entered the module ‘the head’, which includes a course in medical computer science. This course consists of five seminars which are taught in seven groups of about 20 students each and take place in the first five consecutive weeks of this module. Students are randomly assigned to a seminar group at the beginning of the module. For this study, students worked on one of the five newly designed e-learning cases at the end of each seminar, where 30 minutes were dedicated to working with each case. Cases were provided in the order mentioned above. Every week, the students worked on a case either by themselves or with a partner (about 50% of the students from each group had to work with a partner due to the number of computers available for each group), whereby many alternated between solo and teamwork. Participation was voluntary and anonymous. A member of the Ethics Committee of the Chamber of Physicians, Hamburg, confirmed the innocuousness of the research protocol and written informed consent was obtained from participants. Before working with the first case, students filled out the introductory survey. After each case, a feedback sheet was completed and after the fifth case, the final survey and the multiple choice questions were answered.

### Statistical analysis

Only students who handed in the introductory survey, answered the multiple choice questions and completed at least three case feedback sheets were included in the analysis. This selection was made because in order to receive credits for this course it is mandatory to attend at least three seminars. For comparison, participants with complete data sets were dichotomised with respect to the information about their prior knowledge, namely students in their first clinical semester and students in higher clinical semesters. This was done to account for their different perspectives and skills. For evaluation of the multiple choice test, the scores were divided into five groups (one for each case) with three questions each. To estimate the relationship between the different case rating aspects and teamwork as well as the three case design characteristics narrative style, question type and question content fitted mixed model was used while these predictors were modelled as fixed effects. For adjustment of the cluster structure, resulting from the multiple measurements, every student rated at least one VP case and every VP case was rated by several students. Both clusters were modelled as crossed random effects. Further variables of interest or potential confounders, which were also modelled as fixed effects were students’ gender, age, previous knowledge, e-learning interest and e-learning experience. Additionally, an interaction term between teamwork and previous knowledge was included and excluded when not significant. Aspects from the final survey were analysed by regression analysis with the same list of predictors used for the mixed model analysis. Adjusted means and 95%-confidence intervals (95%-CI) are reported. P-values <0.05, two sided, were considered significant. Nominal p-values are reported without correction for multiplicity. All analyses were conducted using Stata 13.1, STATA Corporation, college station, Texas, US.

## Results

Of the 146 students participating in the course, 108 were included in this study. The primary reason for exclusion was not answering the final multiple choice questions, which eliminated all students who did not attend the fifth seminar. Sociodemographic data of the 108 included students and their disposition towards e-learning are provided in Table 3.

**Table 3:** Students’ characteristics with respect to their prior clinical knowledge and their disposition towards e-learning

	**Participants (n)**	**Age mean (SD)**	**E-learning interest mean (SD)**	**E-learning experience mean (SD)**
Prior clinical knowledge	Male (18)	26.16 (4.33)	4.33 (1.23)	3.72 (1.13)
	Female (32)	24.56 (5.49)	3.94 (1.19)	3.31 (1.26)
	Total (50)	25.14 (5.12)	4.08 (1.21)	3.46 (1.22)
No prior clinical knowledge	Male (18)	24.00 (2.52)	4.67 (0.84)	4.00 (1.28)
	Female (40)	22.37 (2.64)	4.15 (1.05)	3.45 (0.93)
	Total (58)	22.88 (2.67)	4.31 (1.01)	3.62 (1.07)

A total of 496 case feedback sheets were returned over all five cases. Occasional missing answers led to reduced total numbers for some items. The results of the case feedback sheets and the multiple choice test are summarized in Table 4. The topics of the VP cases were considered to be very relevant (5.1; 95%-CI [4.9;5.2]). Except for “relevance” and “question content”, female students rated all case feedback items (“motivation”, “perceived learning effect,” “narrative style” and “question type”) significantly more positively than male students did. Furthermore, students who had a greater interest in e-learning rated all aspects except “question content” more positive. While students who worked in teams received a significantly higher test score (p = 0.038) they reported a significantly lower perceived learning effect compared with students without teamwork (p = 0.046). Students with prior clinical knowledge also received better test scores (p < 0.001). Students with prior clinical knowledge or with e-learning experience needed significantly less time to work with the VP cases (p < 0.001 and p = 0.047, respectively) and students who worked in teams were occupied with the VP cases significantly longer (p < 0.001).

**Table 4:** Effects of student characteristics on case feedback and MC score

**Variables**		**Relevance mean [95%-CI]**	**Motivation mean [95%-CI]**	**Perceived learning effect mean [95%-CI]**	**Narrative style mean [95%-CI]**	**Question type mean [95%-CI]**	**Question content mean [95%-CI]**	**Minutes spent working mean [95%-CI]**	**MC score mean [95%-CI]**
**Sex**	**Male**	5.1 [5.0;5.2]	4.2 [4.0;4.4]	4.1 [3.9;4.4]	4.4 [4.2;4.7]	4.3 [4.1;4.5]	4.5 [4.4;4.7]	n.s.	
	**Female**		4.5* [4.4;4.7]	4.5* [4.3;4.7]	4.8* [4.6;5.0]	4.7* [4.5;4.8]			
**Prior knowledge**	**No**		n.s.	n.s.	n.s.			14** [12;15]	1.7 [1.5;1.9]
	**Yes**							12 [10;13]	2.1** [1.9;2.3]
**Teamwork**	**No**			4.5* [4.3;4.7]				12 [11;13]	1.8 [1.6;2.0]
	**Yes**			4.3 [4.1;4.5]				13** [12;14]	1.9* [1.7;2.1]
**Age**				n.s.				n.s.	n.s.
**E-learning experience**								-0.5*^1^ [-1;0]	
**E-learning interest**		0.16*^1^ [0.04;0.28]	0.21*^1^ [0.07;0.36]	0.17*^1^ [0.02;0.31]	0.15*^1^ [0.01;0.30]	0.16*^1^ [0.02;0.31]		n.s.	

*p < 0.05 **p < 0.001 ^1^gradient [95%-CI].

The final survey (Table 5) revealed that students with an interest in e-learning or with prior knowledge would like to spend significantly less time with a case (p < 0.019 and p < 0.021, respectively). Students with an interest in e-learning also showed significantly more interest in increased media use for the cases, would work on similar cases in their free time, would like a ‘take’ home message’ at the end of each case and read all ‘answer comments’ thoroughly. Meanwhile they did not significantly differ in having read the ‘experts comments’. Furthermore, students with no previous clinical knowledge and female students read the ‘expert comments’ significantly more frequently (48%; 95%-CI [45%;52%] versus 33%; 95%-CI [30%; 37%] and 48%; 95%-CI [44%; 53%] versus 38%; 95%-CI [35%; 41%]).

**Table 5:** Influence of student characteristics on final evaluation questionnaire

**Variables**		**Ideal case duration mean [95%-CI]**	**More media usage would improve the cases mean [95%-CI]**	**I could imagine working on similar cases outside of class mean [95%-CI]**	**I would like a ‘take-home message’ at the end mean [95%-CI]**	**I read all answer comments thoroughly mean [95%-CI]**	**I read the ‘expert comment’ (%) mean [95%-CI]**
**Sex**	**Male**	n.s.	4.2* [4.1; 4.4]	n.s.	5.0* [4.9; 5.2]	n.s.	48%** [44%; 53%]
	**Female**		4.0* [3.9; 4.1]		4.8 [4.7; 4.9]		38% [35%; 41%]
**Prior knowledge**	**No**	13.5* [13.1; 13.9]	n.s.		4.8 [4.7;4.9]	4.1* [4.0; 4.2]	48%** [45%; 52%]
	**Yes**	12.7 [12.3; 13.2]			5.0* [4.9; 5.1]	3.8 [3.7; 3.9]	33% [30%; 37%]
**Age**		n.s.	-0.03*^1^ [-.05;0]		n.s.		n.s.
**E-learning experience**			0.17*^1^ [0.07; 0.27]				
**E-learning interest**		0.42*^1^ [0.07; 0.77]	0.14*^1^ [0.04; 0.24]	0.27**^1^ [0.1; 0.36]	0.09*^1^ [0.00; 0.19]	-0.10*^1^ [-0.20; 0.00]	

*p < 0.05 **p < 0.001 ^1^gradient [95%-CI].

## Discussion

Students answered significantly more questions correctly when they had worked on a case with a partner. Although this effect is only small, it supports our hypothesis that teamwork increases the retention of medical knowledge from VP cases. It is known from continuing medical education that participants consider team-based case discussions to be important to enhance their learning [13]. Interestingly, in our study students perceived a significantly higher learning effect when they worked by themselves even though their test scores demonstrate the opposite. It is possible that the level of difficulty might not have been appropriate for students with less clinical experience, due to the presentation of too much new information at once [12]. This overload of new information might also have led to a more superficial learning approach [18]. However, when interpreting our results and comparing them with the study by Huwendiek et al. [12], it needs to be taken into account that our study was performed in a medical computer science course and the VPs were not embedded in a clinical setting. This could lead to certain limitations in the comparison.

In our setting, students with no prior clinical knowledge read the ‘expert comments’ more frequently than students with prior knowledge and needed significantly more time to work on the VP cases. This can easily be attributed to needing more time to interpret the given information and skipping less of the explanations. Students without prior knowledge performed significantly worse on the MC test. This underscores the theory that the activation of prior knowledge, in combination with acquiring new knowledge [18], seems to be a successful learning strategy when working with VP cases. Hence, the design and content of VP cases should be adapted to the placement of the cases in medical students’ curriculum. Another area for improvement could be the placement of relevant information where it cannot be skipped, rather than on optional cards, like the ‘expert comment’, which can be used at the student’s discretion. Skipping information while using VPs has been documented as an issue in other studies as well [19]. Another important factor to prevent students from skipping information is case length. When asked, students in our study wanted to spend only about 13 minutes working on each case. In actuality, students spent about 13 minutes per case. Cases that appear too long may make students inclined to skip over relevant information.

Relevance, a design aspect [12] that could not be modified in our VP cases has been described as one of the five core attributes of a conceptual framework for designing patient cases for medical education in general [20] and is one of the ten principles of virtual patient design [12]. In our e-learning setting, participants considered the content of the VP cases to be very relevant independent of case design, prior knowledge or teamwork. Hence, the topics of our VPs seem to have been chosen well. However, the desire for a take-home message at the end of a VP case to summarize the most important aspects of the case reached the highest score in our final questionnaire. This desire was significantly stronger in students with less prior clinical knowledge. This suggests that Huwendiek et al.’s [12] recommendation for a ‘focus on relevant learning points’ needs further consideration in our case design to reach the greatest learning benefit, especially when we want to use it with students with less clinical experience . Contrary to other findings on team-based learning [13,16], working in teams had no significant effect on students’ motivation to work with VP cases in our study.

Of the three case design principles that we modified in our VP cases, Huwendiek et al. postulate that VPs should require students to make all the clinical decisions a doctor would make [12]. This, however, could be very time-consuming. In our study, students wished to spend an average of about 13 minutes working with a VP case. In fact, they did work for approximately 13 minutes per case, even though the cases were designed to take up to 30 minutes when worked on thoroughly. When students worked in teams or had less clinical knowledge, they spent a significant but practically irrelevant one to two minutes more on each case. While adequate study time should be provided when integrating e-learning modules [21], with respect to our findings, it seems necessary to compromise on the design of VP cases between the authenticity of the students’ tasks, the conveyance of relevant aspects of medical knowledge and the time students might be willing to spend on a VP case. Since students’ ratings for the VP case design criterion ‘narrative style’ did not reveal any significant differences – except for being rated more positively by female students - using a short narrative style for VP cases might be an option to save reading time.

Regarding the use of media, Huwendiek et al. recommend ‘adequate use of media’ to make things as realistic as possible by providing a picture or footage of the patient and specific findings [12]. Although our use of media was less than what Huwendiek et al. postulated to be advantageous [12], the feedback showed that there was only a moderate wish for more media in the VP cases, mostly among students with high e-learning interest or experience. While this makes our findings compatible, a study by Moreno & Mayer [22] concluded that more media in e-learning did not have a significant effect on test results. A learning-realism trade-off has also been described to be beneficial in a recently published qualitative study on virtual patient design, which explored what concepts work, and for what reasons [19].

Furthermore, it has been demonstrated that the choice of teaching strategy (i.e. e-learning being mandatory) had a bigger influence on learning outcomes than intrinsic motivation [23]. Students also worked longer on a case when VPs were newly introduced, but lost interest in working with VPs when the cases were not used in a blended learning approach [24]. Students’ time working with VPs also increased when they were aware that the exam covered the learning objectives embedded in the cases [9]. Therefore, the integration of VPs into a mandatory course – as in our study design, even though students were not aware that an exam was to follow – might be of greater influence on students’ learning than the actual time spent on each case. In general, most aspects of our VP cases were rated significantly more positively by female students and by students with an interest in e-learning, even though these groups did not show significantly better test results. Therefore, in order to make students work with VP cases, student perception should be considered in the design. Curricular planners should take into account when and how to work with VP cases for best learning outcomes, considering the students’ level and applying teamwork.

### Strengths and limitations

One strength of our study is that the students were not explicitly recruited, but normal course participants who had not been fully briefed on the purpose of their tasks. They were neither informed about the exact implications of their feedback from each VP, nor that working individually or in a team was one variable being tested in our study. Unfortunately, this design also means that there was no pre-test or control group and that the feedback is potentially less reflective than it would have been with a group that was told to pay attention to certain design details of each VP case. A major limitation of our study is that the VPs were used within a medical computer science course. Students in such a course will approach the VPs with a very different focus than if the VPs were integrated into a clinical course where students would be much more motivated to learn from them. The students were not aware that at the end of the course they were going to be tested on the medical knowledge contained in the five VPs. Thus, their attention was not geared towards retaining certain information from the VP cases in the way it is while preparing for an exam. Due to our repeated pattern of case presentation and evaluation, there might also have been a sequencing effect diminishing the level of reflection in the case feedback. Furthermore, it is a limitation that the questionnaires were not validated.

The dropout rate of students who did not participate in the MC test after the fifth seminar was 26%, most likely due to the fact that it is only necessary to attend three of the five seminars in order to earn the credit for the course. Despite this, we still had 108 participants whose results could be used for statistical analysis. Unfortunately, the multiple choice test we used in this study had to be very short, considering that it was a surprise and that the time for the test had to be deducted from the seminar. To improve the reliability and to confirm the results from this study, a longer MC test with a higher number of questions per VP case needs to be designed. There may also have been a setting bias for the time students spent working on the cases because the cases were presented at the end of regular seminars. Thus, finishing the case meant being able to leave, which may have caused some participants to work faster or more superficially than they might have under different circumstances.

## Conclusion

Considering that students are willing to spend about 15 minutes on average for working with a VP case, short cases with relevant diseases, strategic placement of important information in prominent places and a take-home message seem to be the most important design aspects. Teamwork and cases adapted to the students’ level seem to enhance knowledge retention and thus should be considered in a blended learning approach. Further studies with VP cases, adapted to these requirements and followed by a more extensive and reliable knowledge test, need to be designed in order to corroborate the findings from this study.

## Competing interests

The authors declare that they have no competing interests.

## Authors’ contributions

All authors have contributed sufficiently to the project to be included as authors: All authors designed the study, FJ and MR acquired the data, SS and FJ performed the statistical analysis. FJ and SH drafted the manuscript. All authors read and approved the final manuscript.

## Pre-publication history

The pre-publication history for this paper can be accessed here:

http://www.biomedcentral.com/1472-6920/14/137/prepub
